# Plasmonic nanostructures for shrinking structured light to access forbidden transitions

**DOI:** 10.1515/nanoph-2021-0658

**Published:** 2022-01-04

**Authors:** Kyosuke Sakai, Hiroki Kitajima, Keiji Sasaki

**Affiliations:** Research Institute for Electronic Science, Hokkaido University, Sapporo, Hokkaido 001-0020, Japan

**Keywords:** plasmonic nanostructure, quadrupole transition, structured light

## Abstract

Plasmonic nanostructures have considerable applicability in light–matter interactions owing to their capacity for strong field confinement and enhancement. Nanogap structures allow us to tailor electric field distributions at the nanoscale, bridging the differences in size and shape of atomic and light structures. In this study, we demonstrated that a plasmonic tetramer structure can squeeze structured light into a nanoscale area, in which a strong field gradient allows access to forbidden transitions. Numerical simulations showed that the gold tetramer structure on a glass substrate possesses a plasmonic eigenmode, which forms structured light with a quadrupole profile in the nanogap region at the center of the tetramer. The top–down technique employed using electron-beam lithography allows us to produce a gap size of approximately 50 nm, which supports plasmonic resonance in the near-infrared regime. In addition, we demonstrated an array architecture in which a collective lattice resonance enhances the intensity of the quadrupole field in multiple lattice units. This study highlights the possibility of accessing multipolar transitions in a combined system of structured light and plasmonic nanostructures. Our findings may lead to new platforms for spectroscopy, sensing, and light sources that take advantage of the full electronic spectrum of an emitter.

## 1 Introduction

Spontaneous emission, a fundamental process in the field of light–matter interaction, is responsible for the characteristic emission spectrum of an emitter. In principle, an excited electron can fall into any unoccupied lower-energy level via this process. However, in practice, the majority of radiative decay channels are too slow to be accessible, rendering most of the spectrum invisible and inaccessible. Among the numerous forbidden light–matter interaction processes, multipolar transitions have been addressed using modern technologies in nanophotonics [1], [2], [3] and structured light [4], [5], [6]. The ability to access these transitions would allow multiplex and broadband spectroscopy and a light-source platform in which a greater portion of the electronic energy level structure of an emitter can be exploited.

In multipolar transitions, the angular momentum (AM) quantum number of the electron changes by more than one unit. The transitions are slow because the wavelength of emitted light (∼10^3^–10^5^ Å) is typically far larger than the size of the atomic or molecular orbitals participating in the transition (∼1–10 Å). Because of this difference in the length scales, the rate of electronic transitions as a function of AM varies by many orders of magnitude, making high-AM transitions invisible in the absorption and emission spectra of an emitter. Recent theoretical and experimental studies have shown that plasmonic nanosystems can squeeze the wavelength of light by a few orders of magnitude, which allows us to access multipolar transitions [7,8].

The AM quantum number of an electron can be changed by one unit by the circular polarization (spin) of light. Furthermore, the AM quantum number of an electron can be changed by more than one unit using structured light, which has extra AM associated with its spatial mode structure. In particular, Laguerre–Gaussian (LG^*l*^_*p*_) beams carry an additional amount of orbital AM, indicated by the quantum number *l*, per photon [9, 10]. An atom at the center of the LG^1^_0_ beam absorbs two units of AM from a single photon – one from the spin and another from the spatial structure of the beam [6] – even when there is no light intensity but only a field gradient. Light-driven transitions between two atomic states occur if the superposition of their charge distributions matches the multipole structure of the excitation field [11]. A dipole transition is driven by an oscillating field, whereas a quadrupole transition is driven by an oscillating field gradient where there is no light intensity [12].

Structured light with extra AM squeezed into nanoscale spaces using plasmonic nanosystems should allow access to multipolar transitions [13]. For example, when rubidium atoms absorb two units of AM from the squeezed structured light in the plasmonic nanosystem, they exhibit an electric quadrupole transition at a wavelength of 911 nm [14].

**Figure 1:**
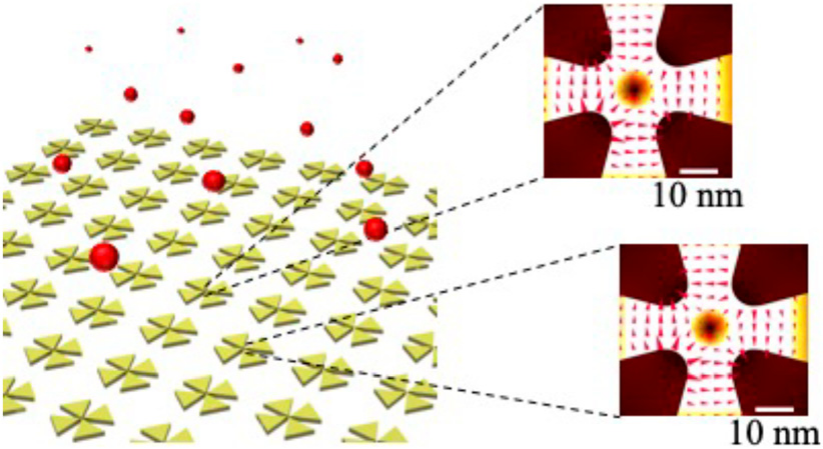
Schematic of the proposed light–matter interaction platform with an array of the plasmonic nanosystem where each tetramer unit exhibits a quadrupolar oscillating field due to collective oscillations of plasmonic resonance.

In the present study, we explored plasmonic nanosystems that can squeeze structured light with extra AM into an area with a diameter of a few tens of nanometers. In particular, we demonstrated that gold tetramer structures exhibit a quadrupolar oscillating field with zero intensity in the central gap region. The structures were designed to have a plasmonic peak at 911 nm, corresponding to an electric quadrupole transition of rubidium atoms. We determined the field distributions and spectral responses of gold tetramer structures using numerical simulations. Moreover, we demonstrated the corresponding experimental spectral response in samples fabricated using electron beam lithography and the lift-off technique. For practical light–matter interaction platforms, we proposed an array architecture with a collective oscillation of plasmon resonance, i.e., a collective lattice resonance, that enhances the intensity of the quadrupole field in multiple lattice units (Figure 1). The concept presented herein of shrinking structured light to allow forbidden transitions provides the possibility of accessing the full electronic spectrum of an emitter, which is critical for a broad spectrum of nanophotonics applications.

## 2 Results and discussion

### 2.1 Single unit of tetramer

**Figure 2:**
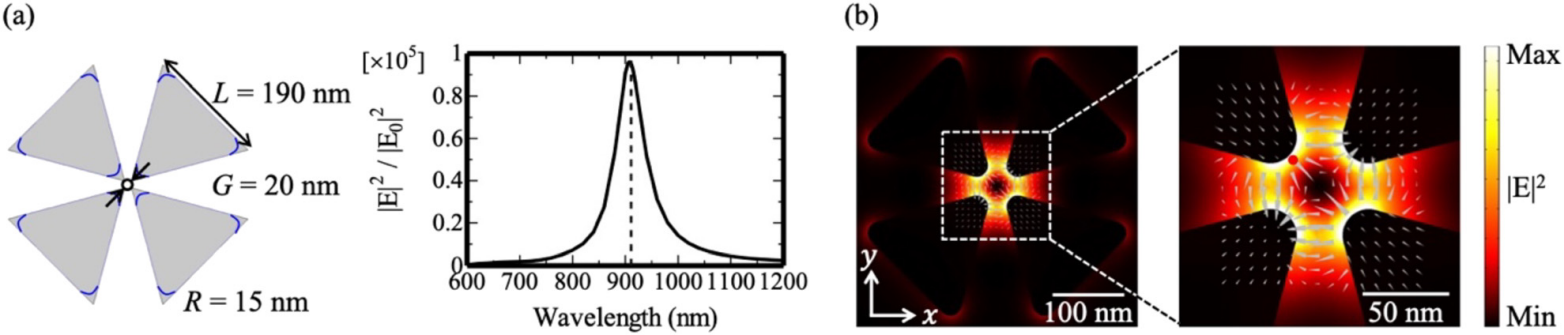
(a) Tetramer unit design and near-field spectrum in the gap region when illuminated by an on-axis cylindrical vector beam. (b) Near-field intensity profile with a snapshot of the electric field vector at 911 nm. The gray triangles indicate the electric field vectors and the red point indicates the position where the spectrum of the near-field intensity was measured.

The plasmonic nanosystem consists of a gold tetramer constructed from nanotriangles The gold tetramer structure was designed such that plasmonic resonance occurs in the near-infrared region (∼911 nm). Four equilateral triangles with side lengths *L* of 190 nm were placed in a cloverleaf arrangement with fourfold rotational symmetry to form a nanoscale gap at the center (Figure 2a). In the simulation model, the tetramer structures were placed on a glass substrate, and the structure dimension parameters were obtained from the average values of the fabricated samples. The spacing *G* between the diagonal triangle corners was 20 nm, and the corners of the triangles were rounded with a curvature *R* of 15 nm. The resultant diagonal distance across the gap was 50 nm, and the thickness of all elements was 30 nm. The spectrum of the near-field intensity (|**E**|^2^/|**E**_0_|^2^) was measured at the hotspot of the tetramer gap region situated 1 nm from the sidewall. All visualizations, including those of electric field distributions, were performed at the plane at the mid-point in the height of the structure. A cylindrical vector (CV) beam [15], [16], [17], a type of structured light with a quadrupole profile [see Subsection 4.1 Numerical simulation, Figure 7b], can be squeezed into the nanoscale gap region because the symmetric nature of the beam cross-section is well-matched to the eigenmode of the localized surface plasmon resonance in this system [13]. The near-field intensity distribution at the peak wavelength (911 nm) depicted in Figure 2b clearly shows that the quadrupole field was successfully squeezed into a nanoscale gap region of approximately 50 nm.

**Figure 3:**
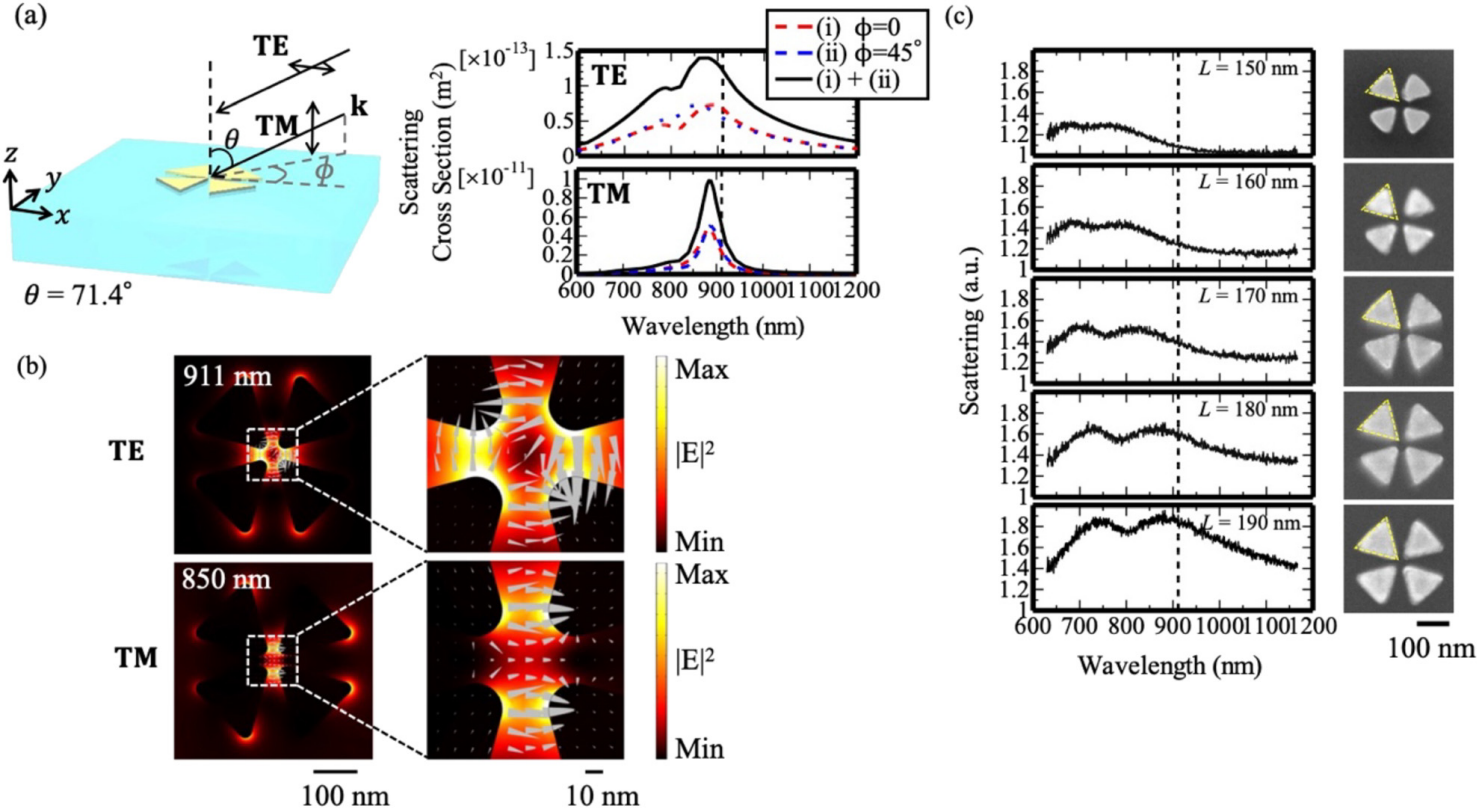
Results obtained under dark-field illumination. (a) Illumination configuration and numerical scattering cross-section spectra. Illumination light beams were incident from two different polar angles: *ϕ* = 0° and 45° with two polarizations (TE and TM). Double-headed arrow indicates the direction of an electric field. (b) Numerical near-field intensity profiles with a snapshot of the electric field vector at 911 nm (TE) and at 850 nm (TM). The gray triangles indicate the electric field vectors. (c) Experimental scattering spectra of the isolated gold tetramer with *L* values of 150, 160, 170, 180, and 190 nm (left) and corresponding SEM images of the fabricated samples (right).

To test the fabricated samples, we compared the scattering spectra of the numerical simulations and the experiment under dark-field illumination, where we can obtain sufficient signals from our nano-structures. Because the formation of the CV beam is highly difficult under dark-field illumination, the sample was illuminated by white light with a large incidence angle (*θ* = 71.4°) and random polarization (Figure 3a). In the simulation, the electric field vector was set parallel (TE) and perpendicular (TM) to the glass surface, and the spectra obtained using two different incidence angles for the illumination light beam were summed to obtain the scattering cross-section spectrum. The scattering cross-section spectrum exhibited a pair of overlapping peaks, with a longer tail at 911 nm. The shorter wavelength peak originated from a localized surface plasmon mode of a single triangle, while the longer wavelength peak originated from hybridized modes in a gap region (dipole mode at 850 nm and quadrupole mode at 911 nm). The numerical simulation revealed that even under this condition, the incident light can excite the quadrupole field in the gap region at 911 nm (Figure 3b). Figure 3c shows the experimental results, specifically, the scattering spectra and corresponding scanning electron microscopy (SEM) images of the samples with different side lengths *L*. The double-peaked spectra underwent redshifting as *L* was increased. The experimental spectra of samples with *L* values of 180 and 190 nm were in good agreement with the corresponding simulated scattering cross-section spectra, which suggests that these samples produced a quadrupole field in the gap region at 911 nm.

### 2.2 Array of tetramers for collective lattice resonances

When metal nanoparticles are arranged in an ordered array, for example, one wavelength apart from each other, they diffract light waves in the plane of the array over a wide area, causing electric field enhancement [18], [19], [20]. In particular, for weak interactions such as multipole transitions, field enhancement within a single nanostructure unit and the multiplication of such interactions can both be beneficial. We designed an array architecture of gold tetramers to produce a collective lattice resonance of the quadrupole field [13, 21].

**Figure 4:**
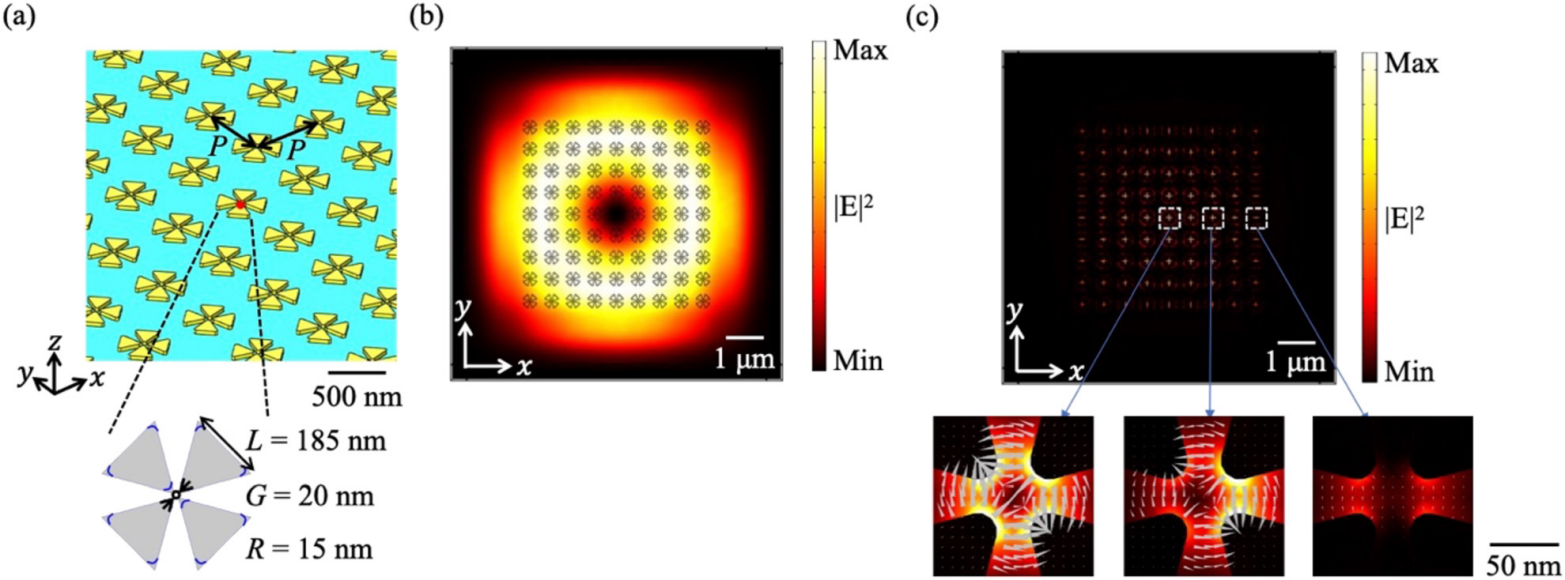
Array of gold tetramers under CV beam illumination. (a) Configurations of the array system. (b) Electric field intensity distribution of the CV beam in the absence of the gold tetramer array. The black overlaid tetramer outlines indicate the positions of the tetramers for the array with *P* = 560 nm. (c) Numerical near-field intensity profile. The lower plots show snapshots of the electric field distributions in which the gray triangles indicate the electric field vectors.

We performed numerical simulations to optimize the structural parameters to obtain the collective lattice resonance of the quadrupole field. Figure 4a shows the array configuration employed in this study; multiple tetramer units were placed in a square lattice arrangement on a glass substrate. The size of the tetramer unit was slightly modified from the single-unit case; moreover, the period *P* was set to 560 nm so that the near-field resonant spectrum peaked at 911 nm. Figure 4b shows the cross-sectional intensity profile of the incident CV beam with a quadrupole profile in free space. The black lines indicate the positions of the gold tetramers; the entire array area (9 × 9) was covered by the incident beam. Figure 4c shows the near-field intensity distribution at 911 nm; the single-lobed envelope function peaked at the center despite the ring-shaped intensity profile of the incident beam. In a finite-sized array, the envelope function has both fundamental and higher-order modes [22]. Figure 4c indicates that the CV beam excited the fundamental mode of the envelope function, that is, a collective lattice resonance in this array structure. The electric field vectors depicted in the plots below the main plot show that the quadrupole field was squeezed into a nanoscale gap region within multiple lattice units.

**Figure 5:**
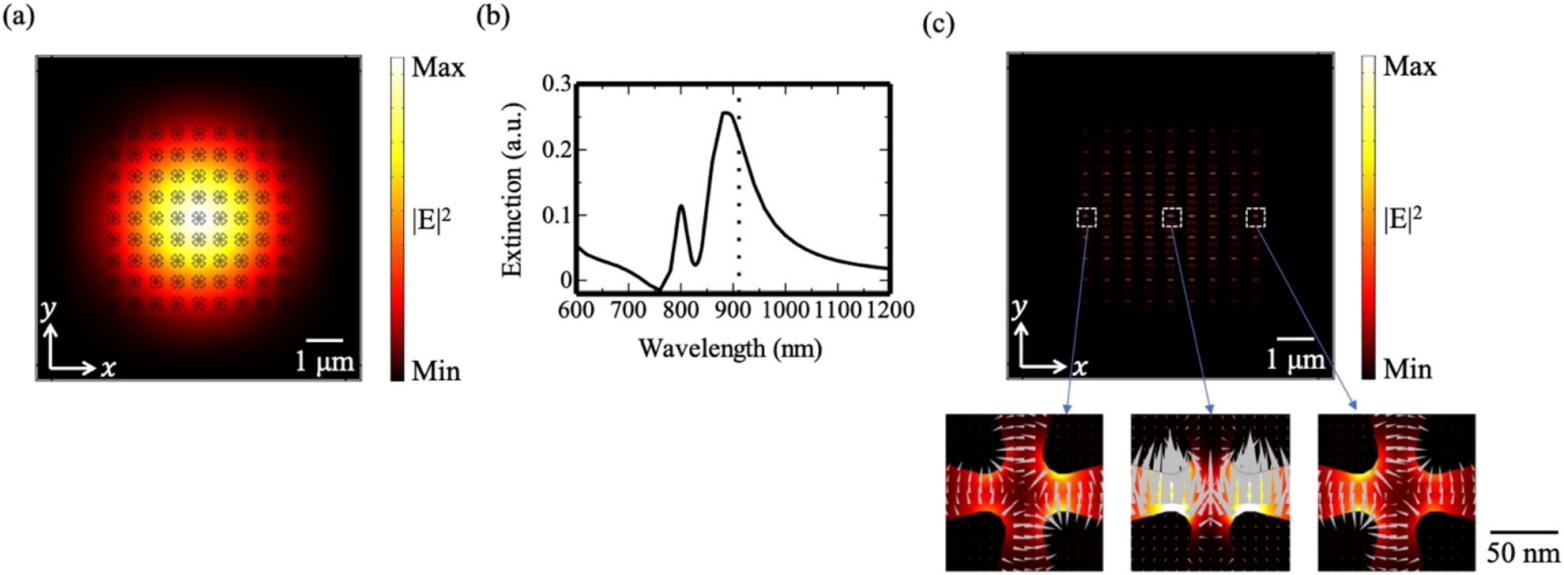
(a) Electric field intensity distribution of the incident Gaussian beam without the gold tetramer array. The black overlaid tetramer outlines indicate the positions of the tetramers for the array with *P* = 560 nm. (b) Extinction spectrum of the gold tetramer array. The black dotted line indicates the wavelength of 911 nm. (c) Calculated near-field intensity distribution of the gold tetramer array at 911 nm. Each of the lower plots shows a detailed view of the gap region inside the white dotted box. The gray triangles indicate electric field vectors.

To test the fabricated samples, we compared the extinction spectra from the numerical simulations and experiments. Although it is preferable to use the CV beam for illumination, the sample was illuminated by a linearly polarized Gaussian beam as the Gaussian beam was a practical choice for experimental use, and we lacked a sophisticated optical system for forming a high-quality CV beam. Figure 5a shows the cross-sectional intensity profile of the incident Gaussian beam in free space. The black lines indicate the positions of gold tetramers; the entire array area was covered by the incident beam. Figure 5b shows the extinction spectrum, which consists of a pair of overlapping peaks, with a longer tail at 911 nm. The spectral intensity at wavelengths shorter than 760 nm originated from a computational error in our simulation that could not be eliminated. Figure 5c shows the near-field intensity distribution at 911 nm. The lower plots demonstrate that even with Gaussian beam illumination, the quadrupole field can be squeezed into a nanoscale gap region within multiple lattice units. Although it is not apparent from the figure, the eigenmode with a quadrupole field profile has a double-lobed higher-order envelope function along the *x*-axis [21]. As a result, the quadrupole mode was canceled out, and only a small dipole mode was observed in the central column along the *y*-axis, whereas quadrupole fields were produced in the nanoscale gap regions within other multiple lattice units.

**Figure 6:**
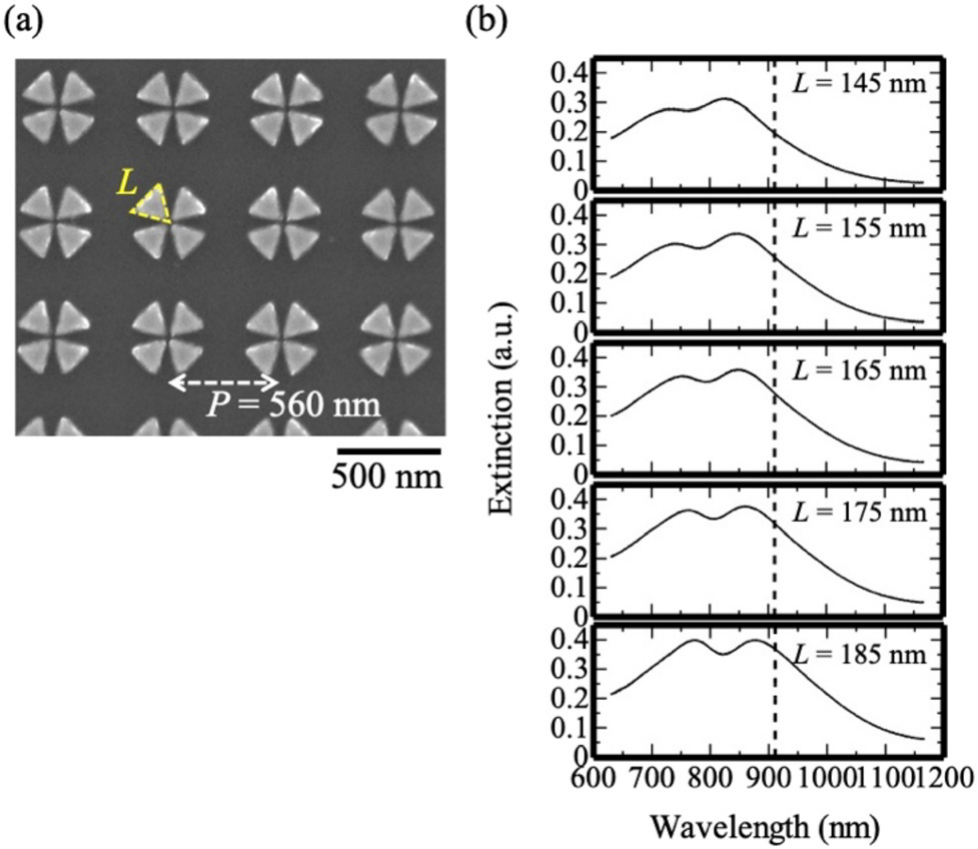
(a) SEM image of the fabricated gold tetramer array. The dimensions are as follows: *L* = 185 nm and *P* = 560 nm. (b) Extinction spectra of the gold tetramer array with *L* = 145, 155, 165, 175, and 185 nm from the top. The black dotted line indicates the wavelength of 911 nm.

Figure 6a shows an SEM image of a sample consisting of a square lattice array of gold tetramers fabricated using the procedure used for the single unit. To observe the array structure using an optical microscope, a square-shaped array with a length of 50 μm was prepared, which was far larger (90 × 90) than that in the numerical simulation (9 × 9). We illuminated the array area using a focused Gaussian beam, with an iris in the confocal plane to ensure that only the transmitted light from the array was collected. The extinction spectrum exhibited a pair of overlapping peaks, with a longer tail at 911 nm (Figure 6b), thus indicating that the fabricated array sample produced quadrupole fields in multiple units at 911 nm. Similar to the trend observed for the single nanostructure unit, the double-peaked spectra were redshifted when the side length *L* of the unit was increased. The discrepancy between the experimental and numerical extinction spectra could be attributed to the difference between the illumination area size and computational error at wavelengths shorter than 760 nm.

## 3 Conclusions

In this report, we presented a plasmonic nanostructure consisting of a clover-leaf-shaped tetramer in which structured light with a quadrupolar profile can be squeezed within an area with a diameter of several tens of nanometers. The plasmonic peak wavelength can be tuned in the near-infrared regime by changing the size of the tetramer. Numerical simulations showed that the plasmonic eigenmode with a quadrupole profile can be excited using a light beam with a large incidence angle in a single tetramer, a linearly polarized Gaussian beam in an array structure, and a cylindrical vector beam. As a realistic light–matter interaction platform, we demonstrated a square-lattice array of tetramers that produces a collective lattice resonance that enhances the field intensity in multiple lattice units. The employed standard top-down technique using electron-beam lithography allowed us to fabricate gold tetramer structures with optical characteristics that are in agreement with those predicted by numerical simulation. The minimum size of the tetramer gap was limited by our fabrication technique, but other techniques are expected to facilitate the superseding of this limit to further squeeze structured light with a quadrupolar profile.

In future studies, as a proof-of-concept, the electric quadrupole transition of the rubidium atom should be observed. For example, when the ^87^Rb atom in the 5p_3/2_ state is placed in the center of a squeezed quadrupolar field, observation of an electric quadrupole transition (5p_3/2_ → 6p_3/2_) and decay directly into the 5s ground state with the emission of a 420-nm photon should be possible [14]. Although we presented a specific tetramer design optimized for 911 nm, our concept based on the extra AM of light in a squeezed field is potentially applicable to a broad range of nanophotonics research and development – including spectroscopy, sensing, and light sources – for which the exploitation of conventionally forbidden light–matter interactions such as high-order multipolar transitions is essential.

## 4 Methods

### 4.1 Numerical simulation

**Figure 7:**
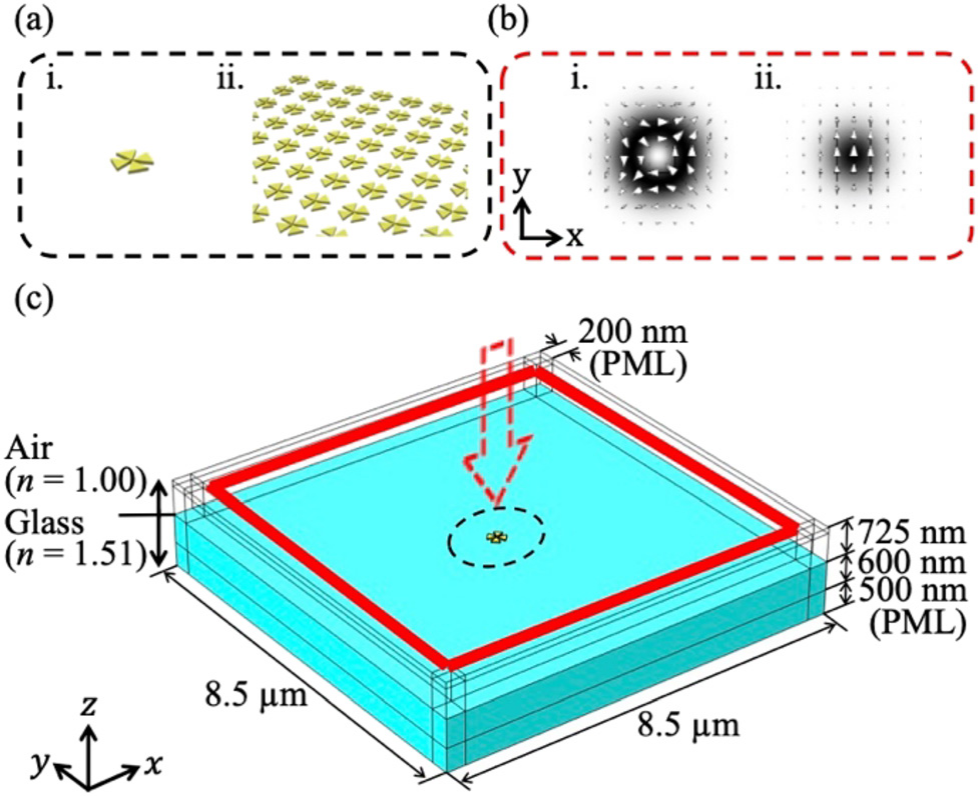
Numerical simulation model. (a) Two types of nanosystems: (i) an isolated gold tetramer and (ii) a gold tetramer array. (b) Schematic of two types of incident beams: (i) a CV beam with a quadrupole profile and (ii) a Gaussian beam with linear polarization. The arrows indicate electric field vectors, and the gray shading indicates the electric field intensity. (c) Geometry of our calculation model considering the gold tetramer structures on the glass substrate in air. The incident beam propagates in the *z*-direction from the top surface (bounded by the solid red lines).

To design our plasmonic nanosystem and simulate its optical properties, we calculated the electromagnetic field in the system using the finite-element method (FEM) in COMSOL Multiphysics. We used two types of nanosystems – an isolated tetramer and a square lattice array of tetramers (Figure 7a) – and two types of illumination light beams – a cylindrical vector (CV) beam with a quadrupole profile and a Gaussian beam linearly polarized in the *y*-direction (Figure 7b). The CV beam was ideal for the efficient excitation of the squeezed quadrupole gap field, whereas the Gaussian beam was a practical choice for experimental use. Because of the limited computer resources and finite size of the illumination beams, the calculation model shown in Figure 7c, consisting of a finite system, was used. This consisted of an isolated gold tetramer or a gold tetramer array (9 × 9) was placed on a glass substrate in air [21]. A perfectly matched layer (PML) formed the side and lower boundaries. The refractive indices of air (*n*_air_) and the glass substrate (*n*_glass_) were 1.00 and 1.51, respectively. The complex refractive index of gold was obtained from a report by Johnson and Christy [23]. We set the complex electric field of the focused incident beam at the top surface (outlined by the solid red lines). The excitation beam propagated downward, forming a beam waist inside the system. All visualizations, including those of electric field distributions and electric field vectors, were generated at the plane marking the mid-point in the height of the structure.

### 4.2 Sample design

The gold tetramer structure was designed such that plasmonic resonance occurred in the near-infrared region (∼911 nm). The structure dimension parameters (*G*, *R*, *L* and *P*) were determined in the following manner. First, we determined the parameters for a single unit of tetramer. The spacing between the diagonal triangle corner tips (*G* = 20 nm) was the minimum value for which a gap could be successfully obtained in our fabrication protocol. For *G* < 20 nm, the neighboring tips were connected. The corner curvature (*R* of 15 nm) was also the minimum; hence, the diagonal distance across the gap (50 nm) was the smallest attainable value in our current fabrication system. Given these experimental limitations, we tuned the *L* values to 190 nm so that plasmonic resonance occurred in the near-infrared region. Second, we determined the parameters for the array of tetramers. On the basis of these optimized values for the single unit, we maximized the peak intensity of the near-field spectrum of the quadrupole field in the gap region by varying the value of *P*. The process ensured that the array structure produced a collective lattice resonance of the quadrupole field. The peak intensity was maximized with *P* = 560 nm, while the peak wavelength redshifted compared with the single unit case. We found that tuning *L* down to ∼185 nm yielded the optimized condition with the peak wavelength at 911 nm.

### 4.3 Sample fabrication

The gold tetramer structures were fabricated on a glass substrate (thickness = 0.13–0.17 mm) by means of electron-beam lithography (EBL) and metal sputtering. We used a high-resolution EBL system (ELS-7000, Elionix) operating at 100 kV. A conventional copolymer resist (ZEP520A, Zeon Chemicals) was diluted with a ZEP thinner (1:1) and spin-coated onto the substrate at 300 rpm for 3 s and at 4000 rpm for 60 s. The substrate was prebaked on a hot plate for 3 min at 180 °C. EBL was performed at 20 pA. The substrate was developed by successively immersing it in ZED-N50 as a developer at 0 °C for 60 s and in ZMD-B as a rinse for 10 s. After development, a 3-nm-thick Cr adhesive layer was deposited via sputtering (MPS-4000, ULVAC) onto the substrate, followed by the deposition of a 30-nm-thick Au film. Lift-off was performed by successively immersing the sample in ZDMAC, acetone, and ethanol in an ultrasonic bath at 45 °C.

### 4.4 Experimental setup

**Figure 8:**
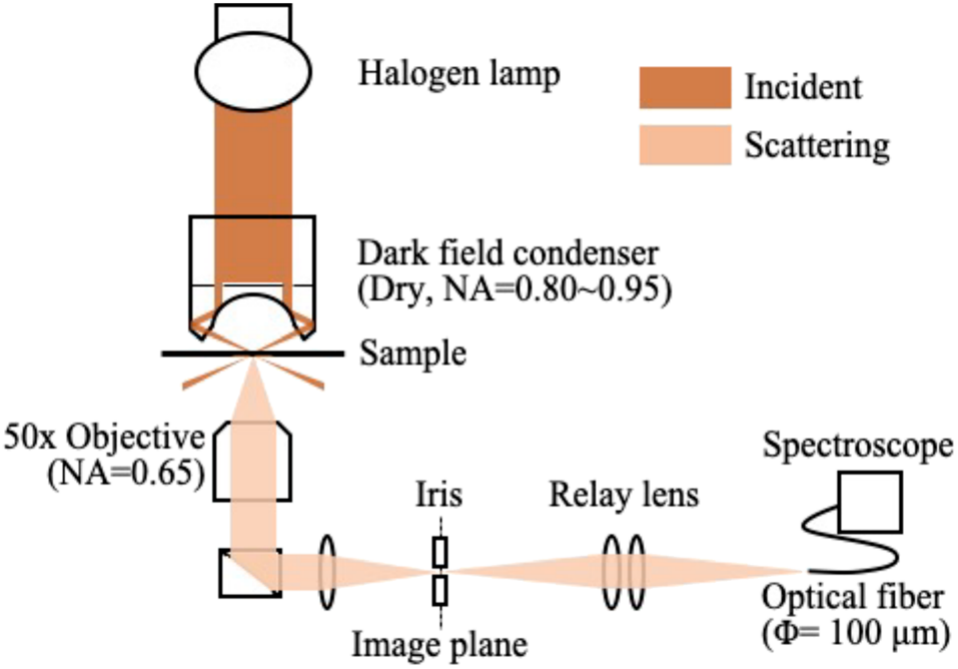
Configuration of the scattering spectroscopy measurement system.

We measured the scattering spectra of isolated gold tetramers using a dark-field illumination microscope system (Nikon Eclipse Ti), as shown in Figure 8. The samples were illuminated by a dark-field condenser (dry, NA = 0.80–0.95), and the scattered light was detected using a 50× objective lens (NA = 0.65). We used a confocal system in which the iris in the image plane cut signals other than those from the gold tetramer structure. To remove the non-uniform incident light spectral intensity distribution, the scattering spectra were normalized to the spectra of the background light obtained for the same sample but without the gold tetramer structure.

**Figure 9:**
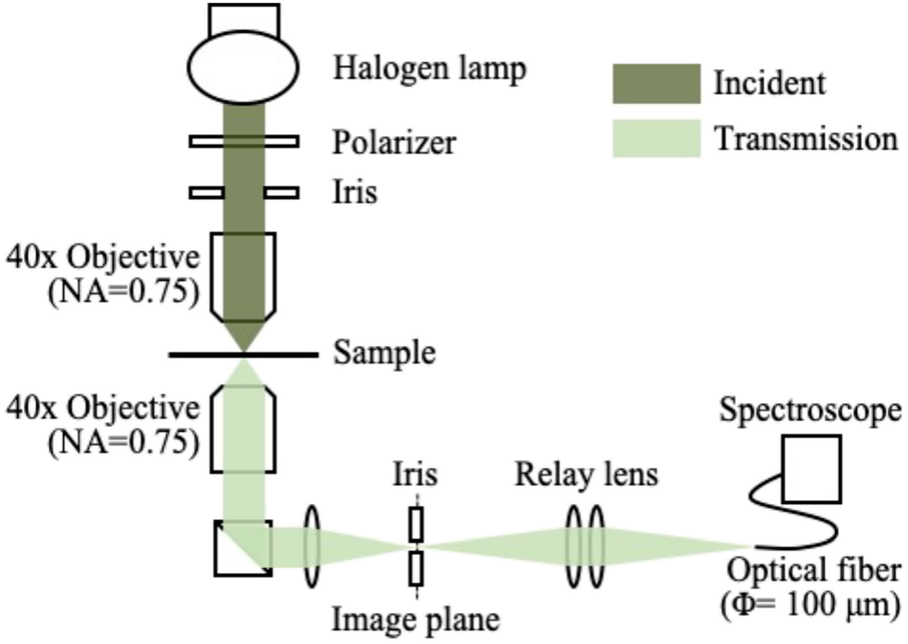
Configuration of the transmission spectroscopy measurement system.

We used the same microscope and confocal system to measure the transmission spectra of the gold tetramer arrays, as shown in Figure 9. Linearly polarized light was focused on the samples by a 40× objective lens (NA = 0.75), and the transmitted light was detected using a 40× objective lens (NA = 0.75). The side length of the gold tetramer array (90 × 90) was 50 µm, which was completely within the cross-section of the focused beam. We used an iris in the confocal plane to ensure that only the transmitted light from the array would be collected. The transmission spectra were normalized to the spectra of light passing through the glass substrate (without any gold structures).
